# A multifaceted field sampling approach for the management of extremely narrow endemic vascular plant species

**DOI:** 10.1002/ece3.9477

**Published:** 2022-11-03

**Authors:** Corrado Marcenò, Alessandro Silvestre Gristina, Salvatore Pasta, Giuseppe Garfì, Leonardo Scuderi, Laurence Fazan, Viviane Perraudin, Gregor Kozlowski, Vito Armando Laudicina, Roberto Venanzoni, Riccardo Guarino

**Affiliations:** ^1^ Department of Chemistry, Biology and Biotechnology University of Perugia Perugia Italy; ^2^ Institute of Biosciences and BioResources National Research Council Palermo Italy; ^3^ IBERIS Società Cooperativa Ribera Italy; ^4^ Botanic Garden and Department of Biology University of Fribourg Fribourg Switzerland; ^5^ Natural History Museum Fribourg Switzerland; ^6^ Eastern China Conservation Centre for Wild Endangered Plant Resources Shanghai Chenshan Botanical Garden Shanghai China; ^7^ Department of Agricultural, Food and Forest Sciences University of Palermo Palermo Italy; ^8^ Botanical Unit, Department STEBICEF University of Palermo Palermo Italy

**Keywords:** ecology, endemic plants, field data collection, microclimate, site conditions, translocation

## Abstract

Extremely narrow endemic plant species (ENEs) are generally connected with microrefugia characterized by particular environmental conditions. In‐depth knowledge of the ecological requirements of ENEs is fundamental to plan appropriate conservation measures. Using cross‐cutting technology, this paper gives a multifaceted approach to collect on‐site data on the ecology of ENEs, defines the protocols for a correct sampling design and describes the type of equipment, the time and expenditure needed. Our sampling approach is based on two orthogonal transects, long enough to extrapolate the whole ecological gradient across the area of occupancy of the target species. Microclimatic data are recorded all along the transects through iButton technology, plus a weather station installed at the intersection of the transects. Microtopographic data are recorded with high‐resolution digital elevation model and sub‐metric GPS. Edaphic data are recorded along the transects through standard soil analyses and on‐site evaluation of the seasonal decomposition rate of organic matter. Additionally, vegetation sampling in 4 m^2^ plots and on‐site germination tests allow to collect data on auto‐ and synecological factors that regulate the life cycle of the target species. Our approach has proved to be cost‐effective and efficient in terms of time spent in the field against the data collected. The most demanding activities were the establishment of the transects and the vegetation sampling. The time spent downloading microclimatic data and testing seed germination was relatively short. Our sampling design allows: (i) to catch as much micro‐topographic variability as possible, both within and out of the tolerance range of the target species, (ii) to minimize the risk of recording identical micro‐topographic conditions compared with a random sampling scheme, and (iii) to ensure quick and relatively easy retrieval of the plots and the equipment both on a multi‐seasonal and multi‐annual basis.

## Graphical abstract

Based on the experience gained from ongoing studies on a Sicilian strictly narrow endemic plant, *Ptilostemon greuteri*, we propose a cost‐effective, multifaceted approach utilizing cross‐cutting technology (data loggers, high‐resolution digital elevation models, etc.) to ensure the on‐site collection of data which can be used to: (i) interpret patterns of occupancy, (ii) monitor demographic variations, such as population numbers, size, density, and age structure as part of the assessment process of the target species, (iii) identify the most suitable sites for translocation, (iv) prepare the actions for its future conservation and (v) to ensure prompt reaction in case of changes that could compromise the survival of the species.

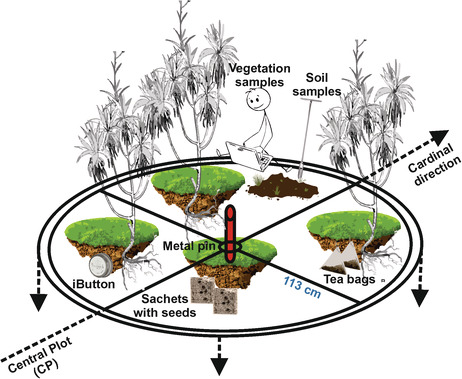

## 1 INTRODUCTION

Extremely narrow endemics (ENEs) usually occur in one or very few populations with a limited number of individuals (López‐Pujol et al., 2013). ENEs occur in climatically diverse regions but generally depend on microrefugia characterized by limited long‐term climatic fluctuations. Microrefugia are often related to small‐scale topographic relief (Dobrowski, 2011) and proximity to persistent warm or mild marine currents (Kier et al., 2009). It is also known that microrefugia act as buffers against climate change (Keppel & Wardell‐Johnson, 2012). However, future climate projections demonstrated that microclimatic refugia could also be prone to climate change (Ohlemüller et al., 2008).

ENEs are often included in regional and national red lists and are the focus of national or international conservation projects (Martín‐López et al., 2009). Although numerous studies have been published on the biology, phylogeny, and demographic trends of these intrinsically rare plant species, a general framework for adequate assessment procedures to model their survival chances is still far from our grasp. For most ENEs, in‐depth knowledge of their ecological preferences and the spatial/environmental boundaries of their microrefugia is still missing.

Without detailed knowledge about the ecological conditions in the growing sites, there is the risk to confuse causes and consequences of the rarity of ENEs. These plants may have such narrow distributions for several reasons: some related to the geologic, palaeoclimatic or evolutionary history of the sites in which they grow, others linked with human intervention such as land‐use or habitat conversion, others yet related to a combination of factors acting locally at different temporal scales (Fiedler & Ahouse, 1992). Understanding the ecology and the environmental functioning of the microrefugia where ENEs grow is therefore fundamental to plan appropriate conservation measures and on‐site interventions to mitigate the impacts of climate change (Keppel & Wardell‐Johnson, 2012).

Based on the experience gained from ongoing studies regarding a Sicilian ENE (Pasta et al., 2022), this paper aims to propose a multifaceted approach utilizing cross‐cutting technology to ensure the on‐site collection of valuable data. We here (i) describe in detail our approach, (ii) discuss the general features a target species should have to fit our approach, (iii) define the protocols for a correct sampling design and (iv) describe the type of equipment, time and expenditure needed.

## 2 DESCRIPTION AND IMPLEMENTATION

### 2.1 Case study

**FIGURE 1:**
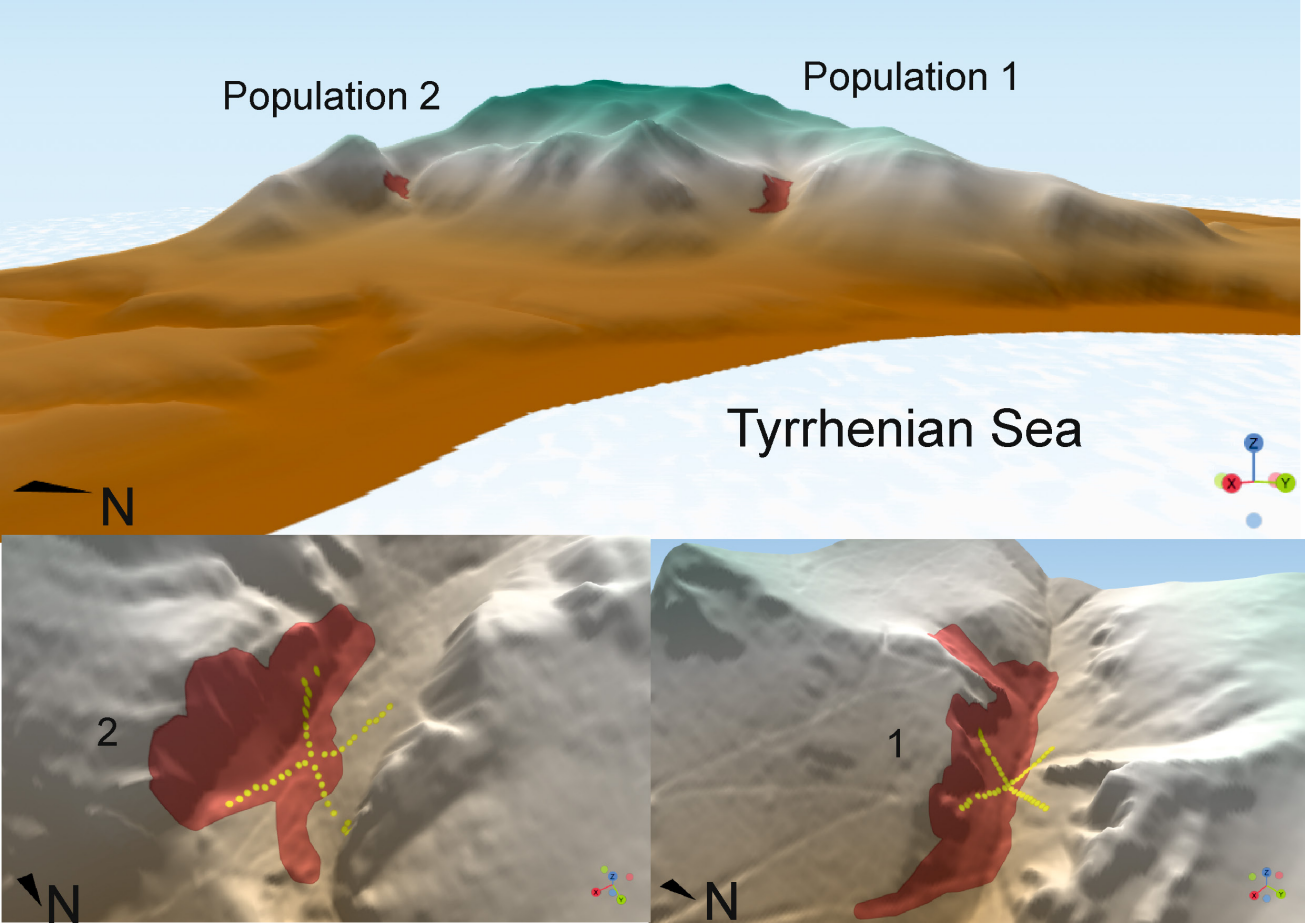
3D view of Monte Inici and details of the sites in which the two populations grow. The area of occupancy (AOO) is in red, and the installed transects are in yellow.

*Ptilostemon greuteri* Raimondo & Domina is a woody herb with large lanceolate leaves (17–20(30) × 2–3 cm), whose traits recall similar cases of woody insular evolution (Zizka et al., 2022). *P. greuteri* is known from only two populations and grows on cliffs, ledges and talus slopes in two NE‐facing canyons of Mount Inici, NW Sicily (Figure 1).

Mount Inici (1066 m a.s.l.) is the north‐easternmost part of the Sicilian‐Maghrebid Foreland‐Thrust belt, mostly made of limestone and dolomitic limestone. Its harsh morphology is shaped by the different responses of the outcropping rocks to the combined effects of past and ongoing karstic and tectonic processes. As a consequence, the whole mountain is furrowed by deep fluvial canyons, sinkholes, caves, and karrens (Catalano et al., 2011).

The climate of the study area is thermomediterranean (Bazan et al., 2015) with an annual mean temperature of 17°C and annual mean rainfall of 595 mm, with 4 months of drought between May and September (data from Castellammare del Golfo, 15 m a.s.l., www.sias.regione.sicilia.it).

*Ptilostemon greuteri* only occurs in narrow canyons, whose aspect allows both populations to benefit from the shade provided by cliffs for most of the day. Both populations also benefit from occult precipitation: moisture rising from the sea condensates very frequently along the northern flank of Mt. Inici, due to the steep thermal landward gradient, locally buffering the summer drought.

### 2.2 Sampling design

**FIGURE 2:**
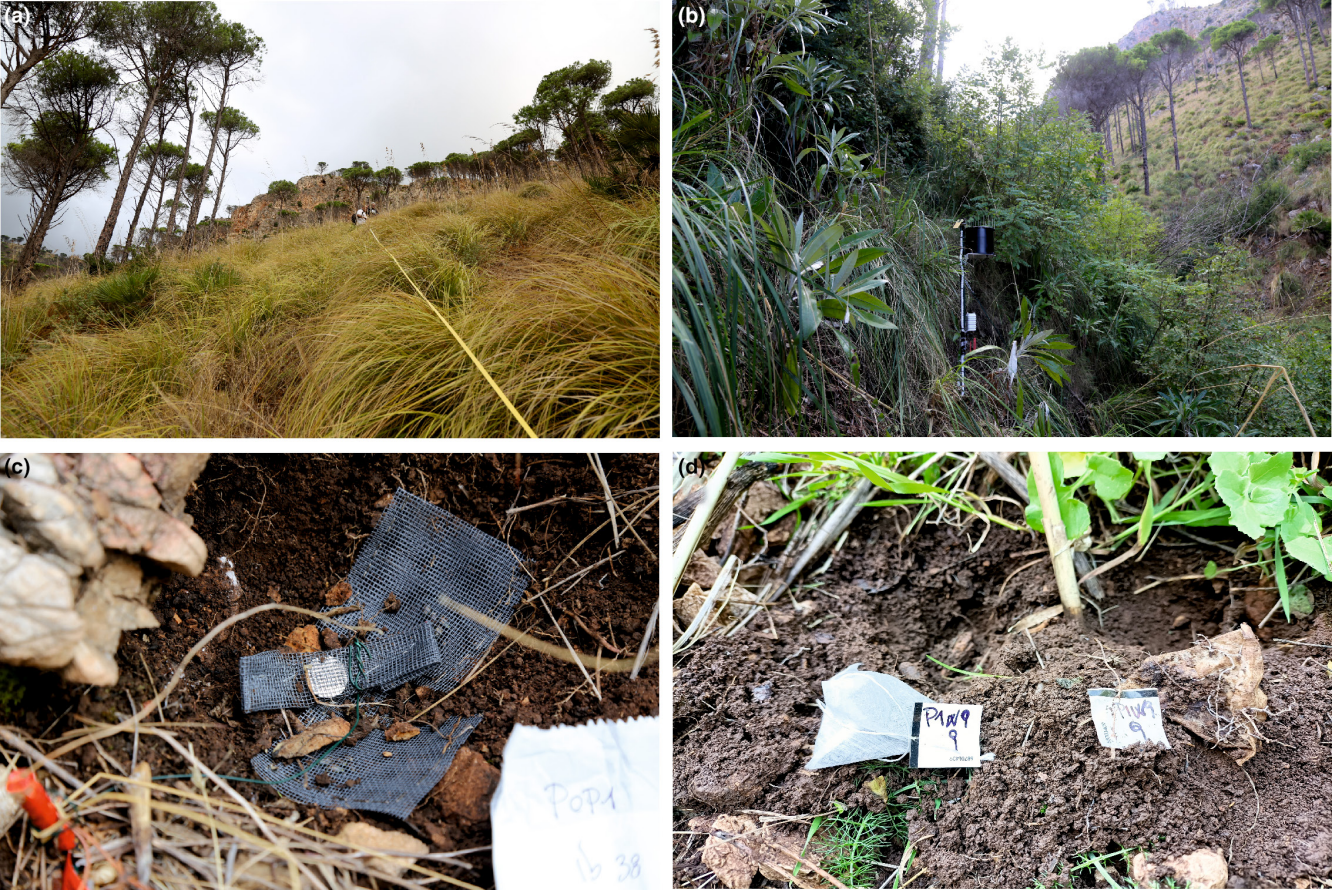
(a) Part of the branch of a transect, (b) weather station, (c) data logger and sachets with seeds, and (d) tea bag replacing.

Our sampling approach is based on two orthogonal transects. In each population, we visually selected a central plot (CP) defined as: the vegetation patch with high percentage cover of the target species, nearest to the center of its area of occurrence (AOO; Gaston & Fuller, 2009). In this way, one can ensure that the CP falls with reasonable approximation within the ecological tolerance of the species (Shelford, 1931). From each CP, following the cardinal directions (N–S and E–W), we established two transects long enough to exceed the AOO of the target species, in order to extrapolate the whole ecological gradient across the AOO. In our case study, each of the four branches of the transect was 100 m long (Figure 1). In each branch of the transect, 4 m^2^ plots were placed every 10 m. Two operators were needed to establish the plots along the transect: the first operator, standing in the CP with a compass, directed the second operator to move along each cardinal direction with a measuring tape, in order to fix the center of the plots along the transect (Figure 2a). The central point of each plot along the transects was marked by metal pins 40 cm long, half driven into the ground. If the center of the plot occurred on a rock, we used spray paint to mark it and the pin was fixed in the nearest proximity. Additionally, each plot was geo‐referenced using a GPS with sub‐metric accuracy (Trimble Geo7X).

### 2.3 Microclimatic data

Since both populations remain in the shade for most of the day, we presumed that microtopographic and microclimatic conditions were highly correlated and that the combination of both allowed the survival of the species by reducing transpiration loss during summer drought. In order to measure the variation of microclimatic conditions, iButton technology (DS1923 Hygrochron, MSI Ltd) was used to record soil temperature and moisture. The iButtons were buried at a depth of 10 cm in the center of each plot along the transect, except for the CP, where a weather station (WatchDog 1650 Series Weather Station, Spectrum Technologies Inc.), was installed in each of the two populations. The iButtons were attached to the metal pin marking the center of each plot in order to facilitate their retrieval. The weather station installed in the CP recorded rainfall, air temperature, soil moisture at two different depths (10–50 cm), and leaf wetness to quantify the effect of moisture condensation (Figure 2b,c).

### 2.4 Soil sampling

Due to the steep ecological gradients occurring along the transects, we expected significant changes in the soil properties from the shaded plots toward those subject to long‐lasting direct solar radiation. Therefore, we collected substrate cores in each plot to perform standard physical, chemical and biological analyses. Details are reported in Appendix S1 (1. Soil analyses).

Additionally, we investigated the decomposition rate of organic matter using the Tea Bag Index (Keuskamp et al., 2013). Following this approach, we used two types of tea bags: Lipton Green and Rooibos Tea. We incubated one pair of bags in the soil of each plot, at a depth of 10 cm, for 90 days. To be sure to catch the seasonal variation in decomposition rates, we extended the incubation experiment for one full year, by collecting the incubated bags and replacing them with new ones every 90 days, starting from mid‐September. Once recovered, the bags were transferred to the lab, air‐dried, cleaned, dried for 48 h at 70°C, and finally, opened to weigh the content. The tea bags were tied to a 40 cm long wooden stick half driven into the ground next to the center of each plot, to facilitate their retrieval (Figure 2d).

### 2.5 Digital elevation model (DEM)

To obtain precise micro‐topographic terrain data and to run the shadow analysis in SketchUp (DeltaCode®) environment, we used a DEM of 2 m spatial resolution provided by the Sicilian Regional Department of Territory and Environment (http://www.sitr.regione.sicilia.it/geoportale/). SketchUp is a 3D modeling software with a user‐friendly interface, that allows importing geo‐referenced DEM to reconstruct daily and seasonal variations of the shading/insolation in any spatial point with the plug‐in Shadow Analysis. As mentioned above, a sub‐metric GPS with maximum error smaller than the spatial resolution of the raster is fundamental to obtain accurate microtopographic data.

### 2.6 In situ germination test and seed viability

**FIGURE 3:**
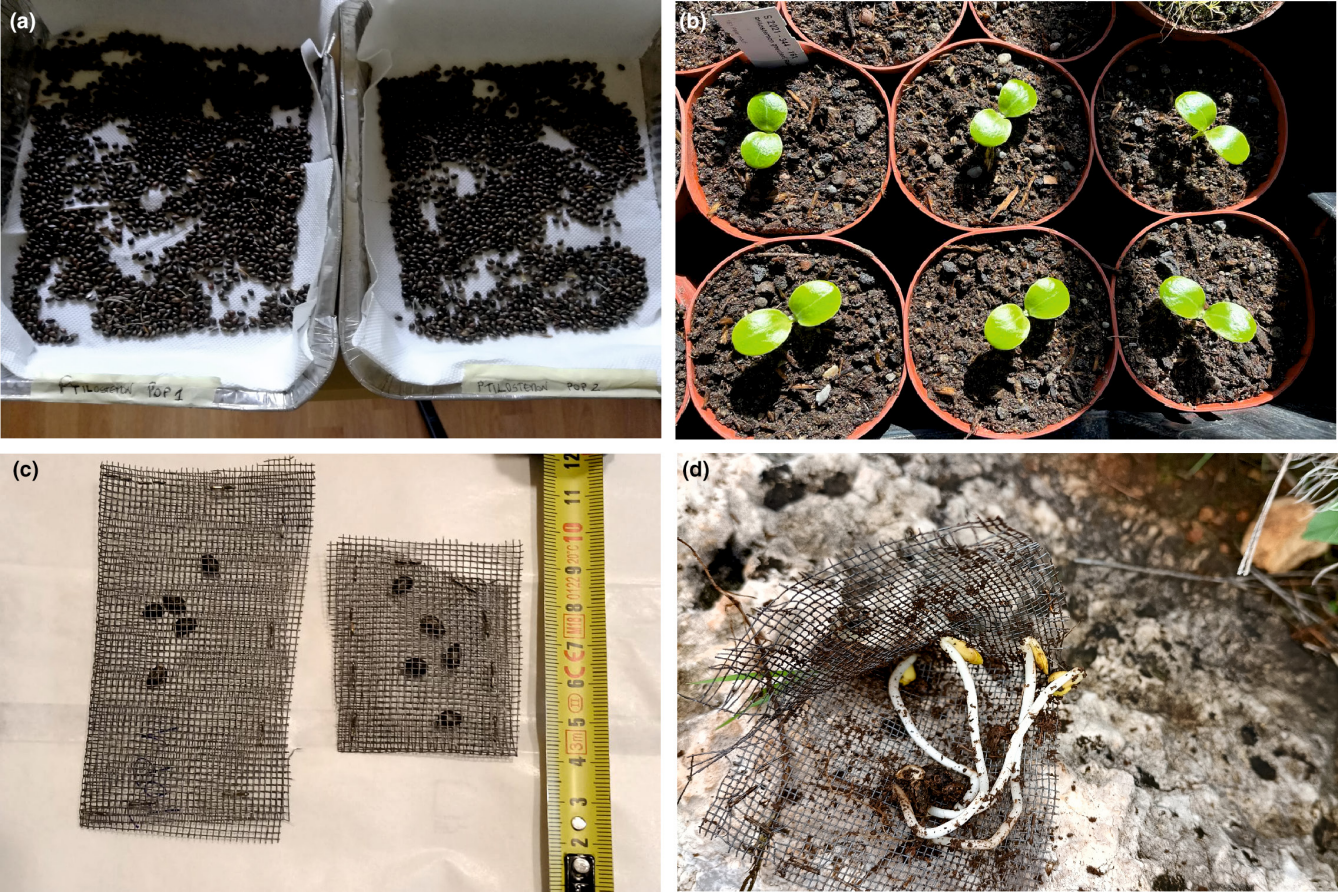
(a) Seed cleaning, (b) seed germination tests/plants production, (c) sachets with seeds, and (d) sachets with germinated seeds.

During spring 2021, we collected ripen seeds of *P. greuteri* in both populations (Figure 3a). The seeds were transferred to the lab, cleaned, and stored in a dry place. One hundred and fifty seeds from each population were used to run standard germination tests (Figure 3b; Pasta et al., 2022). In September of the same year, we prepared 160 sachets made of plastic nets with a 0.5 mm mesh (Figure 3c). Five seeds were put in each sachet and sealed with a stapler. The sachets were transferred to the field and buried to a depth of 10 cm, with two replicates in each plot, making sure that the seeds contained in the sachets came from the same population in which they were buried. To test the germination, every 90 days (coinciding with the replacement of the tea bags), the sachets were dug up to count the number of germinated seeds and reburied in the same position and depth, unless all 5 seeds were germinated (Figure 3d). In which case, the sachet was removed from the plot. After one year, short‐term seed viability was assessed with the tetrazolium test (França‐Neto & Krzyzanowski, 2019), by removing one sachet every two plots, starting from the CP and moving along the transects. The remaining bags remained buried to later assess long‐term seed viability.

### 2.7 Species and vegetation sampling

**FIGURE 4:**
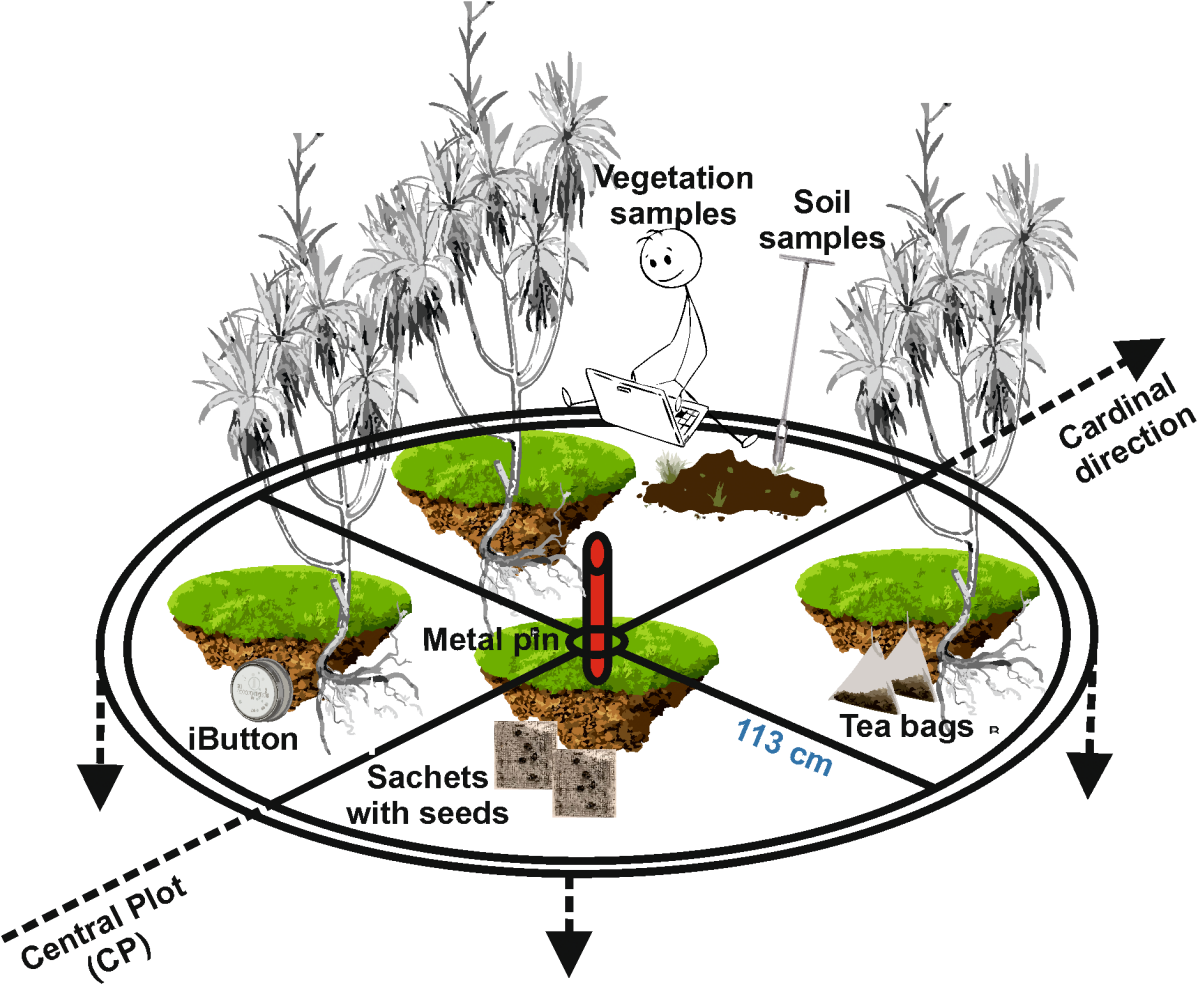
Schematic representation of data sampling in one of the plots along the transects.

In each plot, we collected data at species and community levels (Mucina et al., 2000). We opted for a circular sampling plot of 4 m^2^, corresponding to a radius of 113 cm (Figure 4). The perimeter and four orthogonal spokes of the plot structure were constructed with a flexible, semi‐rigid nylon‐plastic corrugated pipe. The four spokes, converging in the center, were welded to a central hub consisting of a short rigid plastic tube, which was fitted onto the pins marking the center of each plot. Such a flexible, semi‐rigid structure was easily adaptable to the terrain. Having a permanent metal pin in the center of each plot ensures that future surveys will be carried out in exactly the same location. Nevertheless, we also georeferenced the distal points of the four spokes, in order to be able to reconstruct the plot in the future if the metal pins get lost.

Inside each sampling plot, the number of *P. greuteri* plants were counted and their relative abundance was estimated by visual percentage cover. Similarly, vegetation was sampled by recording all vascular plants co‐occurring within the vertical projection of the sampling plot and estimating their relative cover according to the following percentage scale: 0.001, 0.01, 0.1, 1, 5, 10, 25, 50, 75, and 100. The same scale was adopted to estimate the total coverage of the tree, shrub, and herb layers.

## 3 RESULTS

### 3.1 Advantages of the method

Our sampling design was adopted for three main aims: (i) to catch as much micro‐topographic variability as possible, both within and out of the tolerance range of the target species, (ii) to minimize the risk of recording identical micro‐topographic conditions compared with a random sampling scheme, (iii) to ensure quick and relatively easy retrieval of the plots and the equipment both on a multi‐seasonal and multi‐annual basis. It should be noted that we did not lose any equipment or data, apart from two teabags that were probably dug out by a porcupine (*Hystrix cristata* L.).

The standardized approach described here has proved useful for collecting with minimum effort and labor/manpower a large amount of data regarding the site conditions both in the short and in the long term. In the short term, iButtons, soil analyses, rate of organic matter decomposition, and solar radiation provide essential data on the seasonal variability of the site conditions (see Appendix S1: 2. Seasonal and edaphic variability of the site conditions, Figures S1–S5). In addition, the installation of a weather station and permanent plots is essential for the long‐term monitoring of the growing sites of the target species and for vegetation resurveys. Also, time‐series resampling and habitat monitoring can be helpful to provide a framework for developing testable hypotheses about the causes of the rarity and persistence of the target species.

### 3.2 Equipment and time cost

After one year, our approach has proved to be cost‐effective and efficient in terms of time spent in the field against the collected data. Details on the costs and duration of work are reported in Appendix S1 (3. Equipment and working days, Tables S1 and S2). The most demanding activities were the establishment of the transects and the vegetation sampling, both involving one working day for four people in each transect. In our case, the installation of the transects and plots were particularly challenging due to the difficult accessibility of the study area. We worked on slippery grounds with steep slopes, mainly covered by thick vegetation including lianose and thorny species. However, in sites with a less rugged morphology, the installation time could be significantly reduced. Once installed, the time spent monitoring seed germination, collecting and replacing tea bags, collecting soil samples, and downloading data from the weather stations was relatively short, consisting of one working day for two people in each population.

## 4 DISCUSSION

One of the main challenges in conservation biology is to find a cost‐effective field approach, due to the limited financial resources invested in species and habitats conservation (Barbosa & Tella, 2019). Our method optimizes the costs of man labor and equipment in order to obtain multipurpose raw data on the ecological requirements and environmental drivers of narrow‐ranging species. The most significant expense in our protocol is represented by the cost for purchasing iButtons. However, cheaper sensors are also available on the market (Bohan et al., 2018).

On‐site germination data and standard germination tests are also crucial to better understand the strategies that target species adopt to ensure seed recruitment and, eventually, the constitution of a soil seed bank (Fenner, 2012). Standard germination tests are commonly carried out in the lab, under controlled conditions to evaluate the viability of seeds and to assess optimal germination temperature and humidity (Ranal & Santana, 2006). These tests can be done by non‐destructive methods (Xin et al., 2013) and seedlings can be used to produce plants for reinforcement and translocation programs (Abeli & Dixon, 2016). However, on‐site germination experiments are also important, although being seldom run, and mainly for orchids (Rasmussen & Whigham, 1993). The limited distribution range of ENEs makes them ideal subjects for experiments of this kind: the adoption of transects that exceed the AOO of the target species facilitates field data collection on environmental and autoecological factors regulating germination effectiveness and period, seed dormancy and viability. Of course, when dealing with species producing very little amounts of seeds, or represented by only few reproductive individuals, on‐site germination experiments should be carefully considered.

Species and population monitoring programs are remarkably variable and diverse in scale, coverage, and aims (Marsh & Trenham, 2008). The approach proposed here is focused on narrow‐ranging species and can be used to collect a robust initial dataset that will be useful to (i) interpret patterns of occupancy, (ii) monitor demographic variations such as population numbers, size, density, and age structure as part of the assessment process of the target species and to obtain benchmarking values, (iii) identify the most suitable sites for translocation, (iv) prepare the actions for future conservation (Robinson et al., 2018) and (v) ensure a prompt reaction in case of changes that could compromise the survival of the species (Brito‐Morales et al., 2018).

## AUTHOR CONTRIBUTIONS

**Corrado Marcenò:** Conceptualization (lead); funding acquisition (supporting); methodology (lead); writing – original draft (lead); writing – review and editing (equal). **Alessandro Silvestre Gristina:** Conceptualization (equal); methodology (equal); writing – original draft (equal); writing – review and editing (equal). **Salvatore Pasta:** Conceptualization (supporting); methodology (supporting); writing – review and editing (equal). **Giuseppe Garfì:** Conceptualization (supporting); methodology (supporting); writing – review and editing (equal). **Leonardo Scuderi:** Writing – review and editing (supporting). **Laurence Fazan:** Writing – review and editing (equal). **Viviane Perraudin:** Writing – review and editing (equal). **Gregor Kozlowski:** Funding acquisition (lead); writing – review and editing (equal). **Vito Armando Laudicina:** Writing – review and editing (equal). **Roberto Venanzoni:** Writing – review and editing (supporting). **Riccardo Guarino:** Conceptualization (supporting); methodology (supporting); writing – original draft (equal); writing – review and editing (equal).

## CONFLICT OF INTEREST

The authors have no conflicts of interest to disclose.

## Supporting information


Appendix S1

## Data Availability

Regarding the accessibility statement, our work is a methodological paper and we do not have any data to upload to dryad.
