# Supplementary material for: A multifaceted field sampling approach for the management of extremely narrow endemic vascular plant species
Source: Ecol Evol. 2022 Nov 3;12(11):e9477. doi: 10.1002/ece3.9477 (PMC9631324; doi:10.1002/ece3.9477)
Supplement: Supplementary file 1 — Appendix S1 [file ECE3-12-e9477-s001.docx]

**A multifaceted field sampling approach for the management of extremely narrow endemic vascular plant species**

Corrado Marcenò^1*^, Alessandro Silvestre Gristina^2^, Salvatore Pasta^2^, Giuseppe Garfì^2^, Leonardo Scuderi^3^, Laurence Fazan^4^, Viviane Perraudin^4^, Gregor Kozlowski^4,5,6^, Vito Armando Laudicina^7^, Roberto Venanzoni^1^, Riccardo Guarino^8^

^1^Department of Chemistry, Biology and Biotechnology, University of Perugia, Italy

^2^Institute of Biosciences and BioResources – National Research Council, Palermo, Italy

^3^Via Andromaca 60, I-91100, Trapani, Italy

^4^Botanic Garden and Department of Biology, University of Fribourg, Switzerland

^5^Natural History Museum Fribourg, Switzerland

^6^Eastern China Conservation Centre for Wild Endangered Plant Resources, Shanghai Chenshan Botanical Garden, China

^7^Department of Agricultural, Food and Forest Sciences, University of Palermo, Italy.

^8^Department STEBICEF, Botanical Unit, University of Palermo, Italy

* correspondence author: [corrado.marceno@unipg.it](mailto:corrado.marceno@unipg.it)

**1. Soil analyses**

Three soil samples were collected by a shovel from the topsoil (0–15 cm) after removing the surface litter. Field-moist soil samples were divided into two aliquots. The first one was air dried, sieved at 2 mm and stored in sealed polyethylene bottles at 4°C before soil chemical analyses. The second one was freshly sieved at 2 mm, rewetted up to 50% of water holding capacity (WHC), and stored at 4°C before soil biochemical analyses. Soil texture (sand, 2-0.02 mm; silt, 0.02-0.002 mm; clay, <0.002 mm) was determined by the pipette method after shaking soil samples for 2 h and using sodium hexametaphosphate and sodium carbonate (Gee and Bauder 1986). Soil pH was measured in distilled water using a soil/water ratio of 1:2.5 (w/v) and a glass membrane electrode. Soil electrical conductivity (EC) was measured in distilled water using a soil/water ratio of 1:5 (w/v). Total organic C (TOC) and N (TN) were determined on pulverised soil/compost samples by dichromate digestion (Nelson and Sommers 1996) and by Kjeldahl digestion (Bremmer 1996), respectively. Soil available P (POlsen) was determined according to Olsen et al. (1954). Microbial biomass C (MBC) was determined by the fumigation-extraction method (Vance et al. 1987). Moist (50% WHC) soil aliquots (equivalent to 25 g oven-dry soil) were fumigated with alcohol-free chloroform in vacuum desiccators for 24 h in the dark. After removing the chloroform by repeated evacuations, the soil samples were extracted with 0.5 M K_2_SO_4_ (4 K_2_SO_4_:1 g soil, v/w) for 30 min on a horizontal shaker (200 rpm). Unfumigated soil samples were similarly extracted and used as controls. All soil extracts were filtered through Whatman 42 paper and then analysed for organic C by acid dichromate oxidation. Microbial biomass C was estimated as the difference between the organic C extracted from fumigated and unfumigated samples multiplied by a k_EC_ of 2.64. Microbial quotient was calculated as the percentage of TOC present as MBC. Basal respiration was determined by measuring the evolved CO_2_ during 10 days of soil incubation. Briefly, 20 g of soil at 50% of WHC was placed in a 200 mL glass bottle at 22°C, and the cumulatively produced CO_2_ was determined by a gas chromatograph equipped with a thermal conductivity detector after 10 days of incubation. Metabolic quotient (qCO_2_) was calculated as mg CO_2_–C g^−1^ MBC h^−1^ [(mg CO_2_–C cumulated in 240 h (10 days) kg^−1^ soil)/240 h/g MBC kg^−1^ soil].

**2. Examples of seasonal and edaphic variability of the site conditions**

**
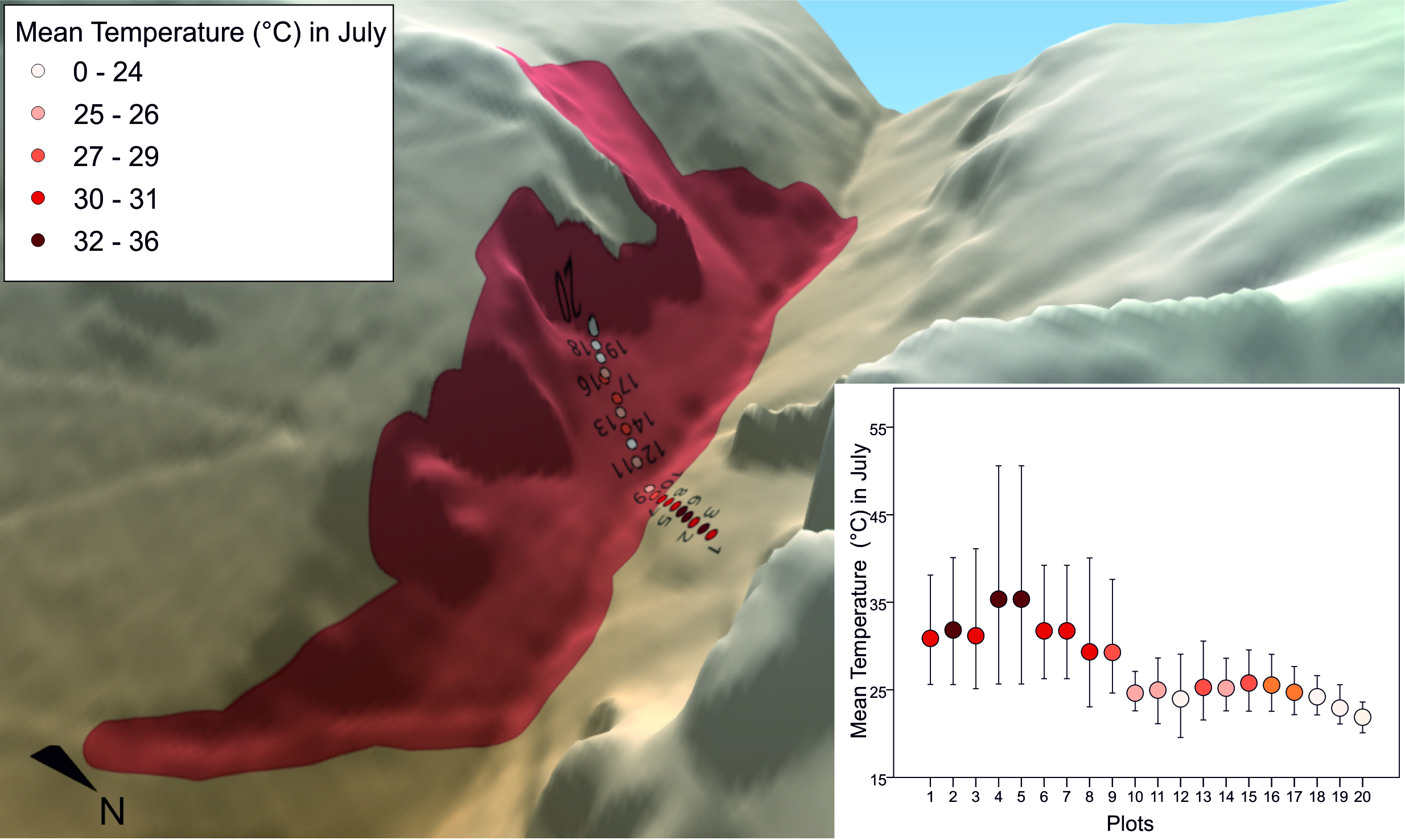
**

**Figure S1. Differences in mean soil temperature of July along the North-South transect in Population 1. The boxplots show the mean, minimum, and maximum temperature in July, recorded in the plots along the North-South transect.**

**
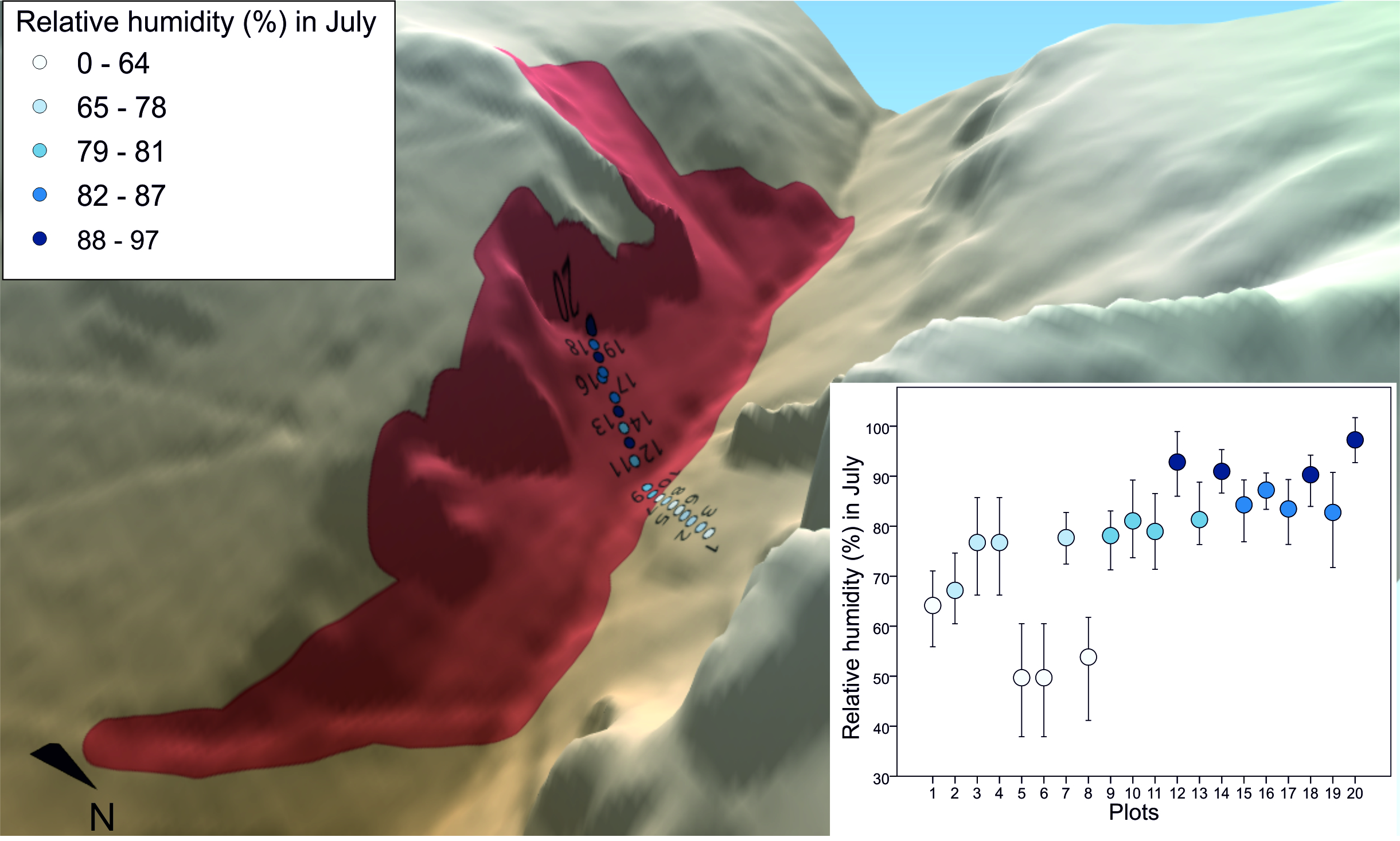
**

**Figure S2. Differences in mean soil relative humidity of July along the North-South transect in Population 1. The boxplots show the mean, minimum, and maximum relative soil humidity in July, recorded in the plots along the transect.**

**
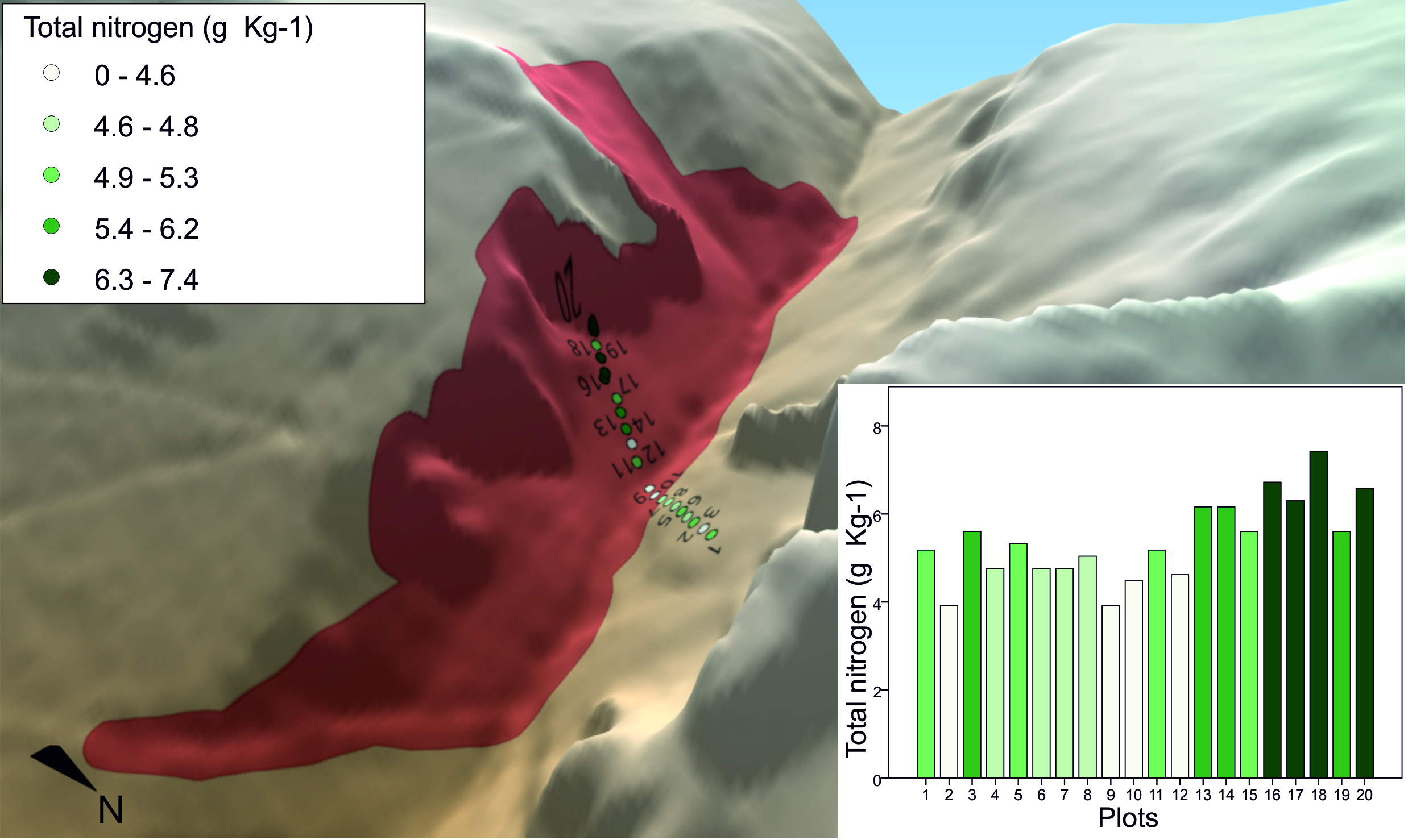
**

**Figure S3. Differences in total nitrogen along the North-South transect in Population 1. The bars show the amount of total nitrogen measured in each plot along the North-South transect.**

**
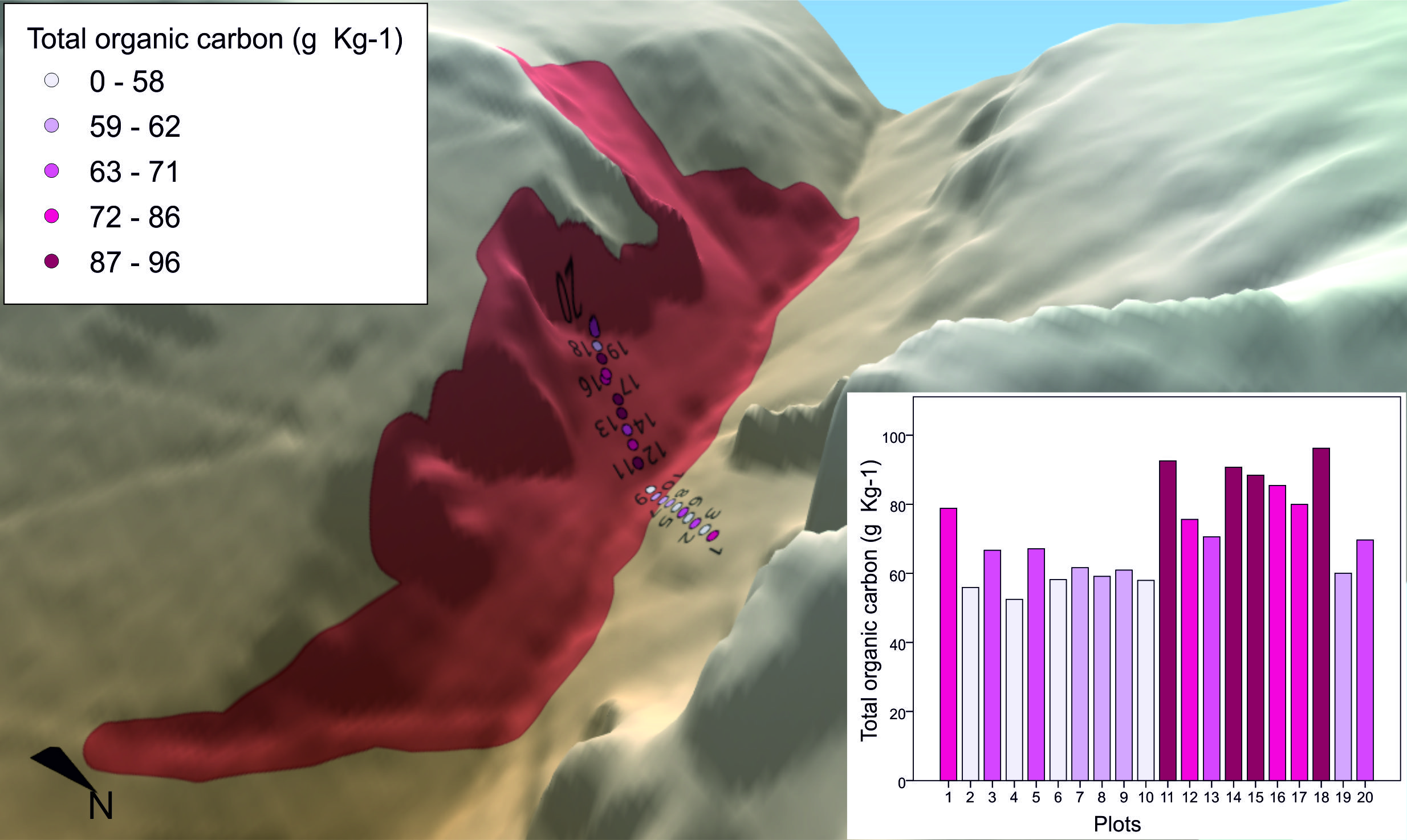
**

**Figure S4. Differences in total organic carbon along the North-South transect in Population 1. The bars show the amount of total organic carbon measured in each plot along the transect.**

**
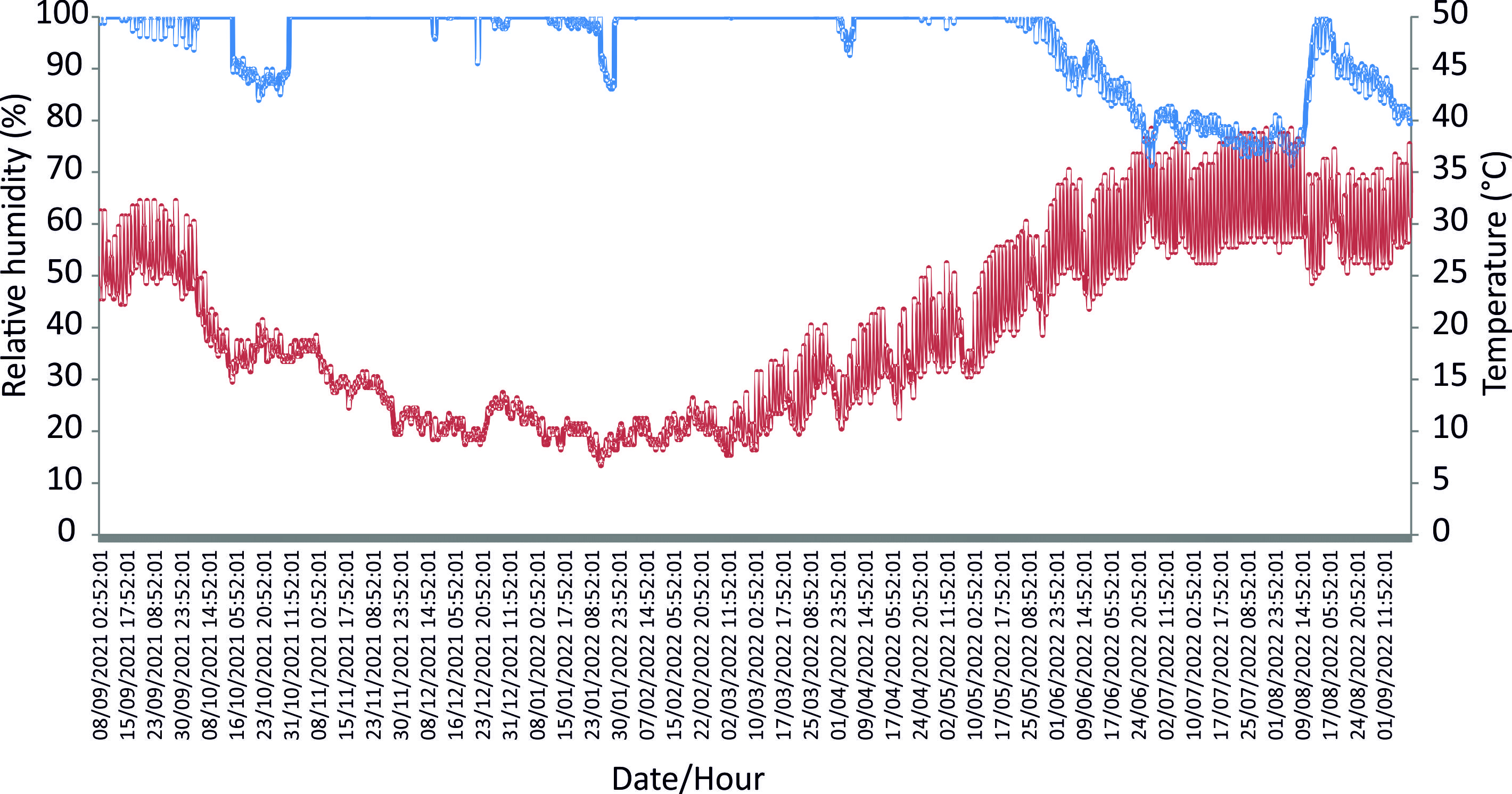
**

**Figure S5. Example of raw data regarding soil temperature (red) and soil relative humidity (blue), recorded by the iButton installed in plot 4 along the North-South transect in Population 1.**

**3. Equipment cost and working days**

| **Items** | **Units** | **Price** |
| --- | --- | --- |
| *Plots setting* |  |  |
| Compass | 1 | €€ |
| Galvanized iron wire (50 m) | 1 | €€ |
| Iron pole for weather station (2.5 m) | 1 | € |
| Mallet | 1 | €€ |
| Measuring tape (50 m) | 1 | €€ |
| Metal pins 40 cm | 50 | €€ |
| Nylon fishing line (100 m) | 1 | € |
| Pickaxe | 1 | €€ |
| Plastic net, 0.5 mm mesh (1 square meter) | 1 | € |
| Pliers | 1 | €€ |
| Shears | 1 | €€ |
| Spade | 1 | €€ |
| Spray paint | 1 | € |
| Sub-meter GPS | 1 | €€€€€ |
| Wooden sticks | 50 | € |
|  |  |  |
| *Microclimate data* |  |  |
| iButton | 1 | €€€€ |
| Weather station | 1 | €€€€€ |
|  |  |  |
| *Soil sampling and analysis* |  |  |
| Carbon steel bucket auger | 1 | €€€ |
| Green tea bags (25 pcs.) | 1 | € |
| Roiboos tea bags (25 pcs.) | 1 | € |
| Shovel | 1 | € |
| Soil analyses (80 samples) | 1 | €€€€ |
| Standard germination tests (250 seeds) | 1 | €€€€ |

**Table S1.** List of the equipment and related costs required for the study of *P. greuteri*. Please note that this is a report of what was used in our experimental approach and not a recommendation of what, with varying capabilities and prices is, available on the market. Also, most of the brands produce a variety of models. Price categories in EUR: € = 1 to 10; €€ = 10 to 50; €€€ = 50 to 200; €€€€ 200 to 600; €€€€€ = 600 to 2000

| **Month** | **Activity** | | **no. of person** | | | | | | **no. of days/person** | | | | | | **no. of working days** |
| --- | --- | --- | --- | --- | --- | --- | --- | --- | --- | --- | --- | --- | --- | --- | --- |
|  | 1 | 2 | 1 | 2 | 3 | 4 | 5 | 6 | 1 | 2 | 3 | 4 | 5 | 6 |  |
| Jun. | Seed collection |  |  |  |  |  |  |  |  |  |  |  |  |  | 2 |
| Jul. | Seed cleaning |  |  |  |  |  |  |  |  |  |  |  |  |  | 1 |
| Aug. |  |  |  |  |  |  |  |  |  |  |  |  |  |  | - |
| Sep. | Setting | Weather station installation |  |  |  |  |  |  |  |  |  |  |  |  | 18 |
| Oct. |  |  |  |  |  |  |  |  |  |  |  |  |  |  | - |
| Nov. |  |  |  |  |  |  |  |  |  |  |  |  |  |  | - |
| Dec. | Tea bags in the field | Tea bags in the lab |  |  |  |  |  |  |  |  |  |  |  |  | 5 |
| Jan. |  |  |  |  |  |  |  |  |  |  |  |  |  |  | - |
| Feb. |  |  |  |  |  |  |  |  |  |  |  |  |  |  | - |
| Mar. | Tea bags in the field | Tea bags in the lab |  |  |  |  |  |  |  |  |  |  |  |  | 5 |
| Apr. | Vegetation sampling |  |  |  |  |  |  |  |  |  |  |  |  |  | 16 |
| May |  |  |  |  |  |  |  |  |  |  |  |  |  |  | - |
| Jun. | Tea bags in the field | Tea bags in the lab |  |  |  |  |  |  |  |  |  |  |  |  | 5 |
| Jul. |  |  |  |  |  |  |  |  |  |  |  |  |  |  | - |
| Aug. |  |  |  |  |  |  |  |  |  |  |  |  |  |  | - |
| Sep. | Removal | Tea bags in the lab |  |  |  |  |  |  |  |  |  |  |  |  | 5 |
| **Total no. of working days** | | | | | | | | | | | | | | | **57** |

**Table S2.** The number (no.) of working days and operators needed during the fieldwork of the study of *P. greuteri*. “Setting” includes plot setting, first tea bags incubation, soil sampling and iButton installation in each plot. “Tea bags in the field” includes tea bags replacement and on site germination check. “Tea bags in the lab” includes drying, cleaning, and weighting of the content. “Removal” includes data downloads from data loggers and removal of tea bags and iButtons. The violet color refers to the number of the operators needed for the first activity, the green one for the second activity. The blu color refers to the number of days spent for the first activity, the orange one for the second activity.

**References**

Bremmer, J. M. (1996). Nitrogen-total. In D. L. Sparks, A. L. Page, P. A. Helmke, & R.H. Loeppert (Eds.), Methods of soil analysis Part 3— Chemical methods (pp. 1085–1121). Madison, WI: Soil Science Society of America, American Society of Agronomy.

Gee, G. W., & Bauder, J. W. (1986). Particle-size analysis. In: Klute A (ed.) Methods of Soil Analysis. Part 1: Physical and Mineralogical Methods. ASA and SSA, Madison, pp 545–567.

Nelson, D. W., & Sommers, L. E. (1996). Total carbon, organic carbon, and organic matter. In D. L. Sparks, A. L. Page, P. A. Helmke, & R. H. Loeppert (Eds.), *Methods of soil analysis Part 3—chemical methods* (pp. 961–1010). Madison, WI: Soil Science Society of America, American Society of Agronomy.

Olsen, S.R., Cole, C.W., Watanabe, F.S., Dean, L.A. (1954). Estimation of available phosphorus in soils by extraction with sodium bicarbonate. U.S. Department of Agriculture, circular 939.

Vance, E.D., Brookes, P.C., Jenkinson, D.S. (1987) An extraction method for measuring soil microbial biomass C. Soil Biol Biochem 19: 703–707.
